# Age Stratification and Stroke Severity in the Telestroke Network

**DOI:** 10.3390/jcm12041519

**Published:** 2023-02-14

**Authors:** Cassie A. Simmons, Nicolas Poupore, Thomas I. Nathaniel

**Affiliations:** 1 Department of Biology, North Greenville University, Tigerville, SC 29688, USA; 2 School of Medicine Greenville, University of South Carolina, Greenville, SC 29605, USA

**Keywords:** age, acute ischemic stroke, comorbidities, telestroke

## Abstract

Background. Age is one of the most important risk factors for stroke, and an estimated 75% of strokes occur in people 65 years old and above. Adults > 75 years of age experience more hospitalizations and higher mortality. In this study, we aimed to investigate how age and various clinical risk factors affect acute ischemic stroke (AIS) severity in two age categories. Methods. This retrospective data analysis study was conducted using data collected from the PRISMA Health Stroke Registry between June 2010 and July 2016. Baseline clinical and demographic data were analyzed for 65–74-year-old patients and those ≥ 75 years of age. *This study aimed to investigate risk factors associated with stroke severity in these two age categories of AIS patients treated in telestroke settings*. Results. An adjusted multivariate analysis showed that the acute ischemic stroke (AIS) population of 65–74-year-old patients experiencing heart failure (odds ratio (OR) = 4.398, 95% CI = 3.912–494.613, *p* = 0.002) and elevated high-density lipoprotein (HDL) levels (OR = 1.066, 95% CI = 1.009–1.126, *p* = 0.024) trended towards worsening neurological function, while patients experiencing obesity (OR = 0.177, 95% CI = 0.041–0.760, *p* = 0.020) exhibited improved neurological functions. For the patients ≥ 75 years of age, direct admission (OR = 0.270, 95% CI = 0.085–0.856, *p* = 0.026) was associated with improved functions. Conclusions. Heart failure and elevated HDL levels were significantly associated with worsening neurologic functions in patients aged 65–74. Obese patients and individuals ≥ 75 years of age who were directly admitted were most likely to exhibit improving neurological functions.

## 1. Introduction

Stroke is a life-threatening and debilitating disease that affects >15 million people each year [1]. Since cardiovascular disease rises with age, older people are more likely to experience strokes, and age is one of the most critical risk factors for stroke [2]. An estimated 75% of all strokes occur in persons aged ≥65 years, and the number of incident strokes is more than double in adults over 75 years [3]. In addition, this age group (>75 years) experiences more hospitalizations and higher mortality [4]. Approximately 50% of all strokes occur in people over age 75, and approximately 30% occur in people over age 85 [5]. Although age categories have independent and differential effects on stroke [6], the risk factors associated with people aged 65–74 years and those older than 75 years have not been well studied. Therefore, there is a lack of accurate management plans to reduce stroke risk factors among older adults in different age categories.

The National Institutes of Health Stroke Scale (NIHSS) score is one of the most widely used tools in stroke neurology [7] to assess neurological deficits after an anterior circulation stroke [8]. It reflects cerebral dysfunction and accesses several clinical issues [9]. In addition, baseline NIHSS scores provide a substantial predictive value for functions after an ischemic stroke [10]. Several interpretations of the NIH stroke scale (NIHSS) have been reported for the classification of stroke severities, including NIHSS scores of 0–6 as mild, 7–16 as moderate, 7–16 as severe, and >16 as very severe [11]. Moreover, NIHSS scores less than or greater than 7 have been used to stratify stroke severity in acute ischemic stroke (AIS) [12,13]. For example, an NIHSS score > 7 provides a prognosis of neurologic worsening of AIS [13], while a score of ≤7 indicates an improving neurological outcome in treated AIS patients [14]. Therefore, baseline NIHSS scores can be used to determine risk factors associated with improving and worsening neurologic functions in AIS patients in an age category when about 75% of all strokes tend to occur [15] compared with an age category when an estimated 50% of all strokes occur and more hospitalization and higher mortality occur [5].

In general, there is even greater difficulty obtaining care that matches current clinical practice recommendations [16]. While intensive stroke management is associated with improved care, with no greater risk of adverse effects [17], many rural physicians have limited access to the resources or technology to offer ongoing support to their patients [18]. Access to subspecialties such as stroke neurologists is also very limited in most rural communities, as is transportation for medical appointments [19]. Therefore, a retrospective data analysis of specific factors that contribute to stroke severity (improving or worsening neurologic outcomes) in the elderly stroke population treated in the telestroke network is an important step to identify comorbidities that can be managed to improve stroke care for male and female stroke patients in the telestroke network. Such an analysis in a telestroke setting in the context of aging, with special considerations for identifying risk factors associated with improving or worsening neurological functions, may help the development of specific management strategies for different age categories of older AIS patients treated in a telestroke network.

Since about 75% of all strokes occur in patients aged ≥65 years, while incident strokes are higher in adults over the age of 75 years [3], this age group (>75 years) experiences more hospitalizations and higher mortality [4]. This provides the rationale to investigate stroke in 65–74-year-old patients compared with those ≥ 75 years old. Therefore, this study aimed to investigate the risk factors associated with stroke severity in these two age categories of AIS patients treated in telestroke settings, and the goal was to identify demographic and clinical factors associated with improving or worsening neurologic functions among older AIS patients treated in the telestroke network. Demographic and clinical risk factors associated with improving or worsening neurologic functions may vary in 65–74-year-old AIS patients, i.e., the age category when about 75% of all strokes tend to occur [15], compared with the age category when an estimated 50% of all strokes occur [5] and more hospitalization and higher mortality occur [4]. Understanding these factors in the telestroke setting could help identify contraindications that could be managed to improve treatment functions among the aging stroke population in the telestroke setting.

## 2. Methods

### 2.1. Statistical Analysis

The primary stroke functions were risk factors associated with improving or worsening neurologic functions in AIS patients aged 65–74 years and ≥75 years treated in the telestroke network. A univariate analysis was used to identify risk factors and differences between the two age categories: 65–74 years old and ≥75 years old. The Pearson χ2 test was used to analyze discrete variables, while Student’s *t*-test was used to analyze all continuous variables. The Pearson χ2 test and Student’s *t*-test were used to determine differences in demographic and risk factors in patients with an NIHSS ≤ 7 (improving neurologic outcome) or an NIHSS > 7 (worsening neurologic outcome) for both the 65–74-year-old and ≥75-year-old age categories. After that, we built binary logistic multivariate analyses by including the established predictors with probability values <0.3 from the univariate analysis. This approach enabled us to capture all predictors approaching significance for the multivariate analysis. Independent predictors of worsening or improving neurologic outcomes in the two groups based on the age categorization were determined. The post hoc adjusted multivariate analysis (logistic regression) determined the demographic and clinical risk factors associated with improving or worsening neurologic outcomes. In the multivariate analysis, the backward selection model approach was used because it allowed all the variables that were approaching significance to be initially included in the model and then removed if they did not add to the importance of the overall model.

The stroke severity based on NIHSS score stratification was the dependent variable in the binary regression model. The primary independent variables were the demographic and clinical risk factors for the groups stratified by age. The odds of presenting with a worsening (NIHSS > 7) or improving (NIHSS ≤ 7) neurologic outcome were analyzed separately for the entire sample independent of age, for the 65–74-year-old group, and for the group aged ≥75 years. The odds ratios and 95% confidence intervals (95% CIs) of the outcome measures were obtained from this model with the significance level set at the probability level of 0.05. These odds ratios were used to predict the independent variables significantly associated with 65–74-year-old and or ≥ 75-year-old groups. The logistic regression model’s sensitivity, specificity, and accuracy were determined using the overall correct classification percentage and the area under the receiver operating curve (ROC). The multicollinearity and the interactions among the independent variables were determined using the Hosmer–Lemeshow test. All analyses were performed using SPSS Version 26.0, and all variables were screened for outliers and univariate normality.

### 2.2. Study Design and Population

The clinical profiles of all patients with AIS registered in the telestroke database of the PRISMA Health Stroke Registry between January 2010 and June 2016 were retrieved and analyzed. The data source for this study was NeuroDirect, a single-hub, rural, 7-spoke, randomized, blinded, controlled trial of a 2-way (hub and spoke hospitals) telestroke network. The NeuroDirect telestroke system used video and teleradiological evaluations of brain scans that allowed bidirectional prognoses [20]. This bidirectional setting allowed the stroke patient to be seen by a stroke neurologist in real time. Under the direct control of a PRISMA stroke expert, the system could move untethered, allowing the stroke neurologist to freely interact with patients, family members, and hospital staff from any of the spoke stations. In addition, a server (PACS) placed in a remote community allowed distant medical experts, such as stroke neurologists and radiologists, to have immediate access to patient CT scans from the spoke hospitals. Interactive advanced 3D visualization and rapid image assessment were conducted using a current-generation device (iPhone, iPad, smartphone, or web browser) without requiring patient image data to be stored on the device. All patients admitted with acute stroke received standard evaluations that included assessments of neurological severity using the National Institutes of Health Stroke Scale (NIHSS) and assessments of disability using ambulatory data, biochemical or laboratory evaluations, the monitoring of vital signs, and all imaging studies during their hospital stays.

The patient data on clinical characteristics, laboratory values, and medical history were all from the stroke registry. The registry has previously been described in other studies [21,22,23]. All variables in this stroke registry had accurate values reported. Data were extracted for age, race, gender, ethnicity, BMI, medication history, and NIH stroke severity (NIHSS) score. Baseline NIHSS data were collected on admission. This was shown in another study to be a marker for the degree of stroke severity [24]. The clinical characteristics or risk factors included atrial fibrillation/atrial flutter, coronary artery disease (CAD), carotid stenosis, depression, diabetes, drug or alcohol abuse, dyslipidemia, a family history of stroke, congestive heart failure (CHF), hormonal replacement therapy, hypertension, migraine, obesity, prior stroke, prior transient ischemic attack (TIA), prosthetic heart valve, peripheral vascular disease (PVD), chronic renal disease, sleep apnea, and a history of smoking.

## 3. Results

A total of 223 AIS patients were identified. In this sample, 104 patients were 65–74 years old, and 119 were ≥75 years old (Table 1). The patients ≥ 75 years old were older; less likely to have a higher BMI (28.99 ± 5.75 vs. 27.22 ± 4.78); and presented with higher rates of atrial fibrillation (28.6% vs. 8.7%), depression (14.3% vs. 5.8%), heart failure (16.8% vs. 7.7%), and previous TIA (16.0% vs. 6.7%) but lower rates of smoking (6.7% vs. 20.2). The ≥75-year-old patients were more likely to be taking antidepressant medication (16.8% vs. 7.7%) and presented with higher initial NIHSS scores (10.63 ± 8.90 vs. 6.58 ± 7.64). In addition, they were more likely to present with higher HDL (45.01 ± 14.09 mg/dL vs. 40.09 ± 10.48 mg/dL), heart rate (80.19 ± 15.69 vs. 75.90 ± 15.53), and NIHSS > 7 (44.7% vs. 31.4%) and were less likely to improve in ambulation after the ischemic event (37.6% vs. 54.3%).

The clinical and demographic characteristics associated with improving or worsening neurological functions were stratified according to the age categories and NIHSS, as presented in Table 2. In 65–74-year-old patients, those with NIHSS > 7 presented with higher rates of heart failure and higher HDL levels. Patients in the age ≥ 75 category with NIHSS > 7 were more likely to be older, less likely to take a cholesterol reducer, and differed significantly in ambulation on admission and discharge.

**Figure 1:**
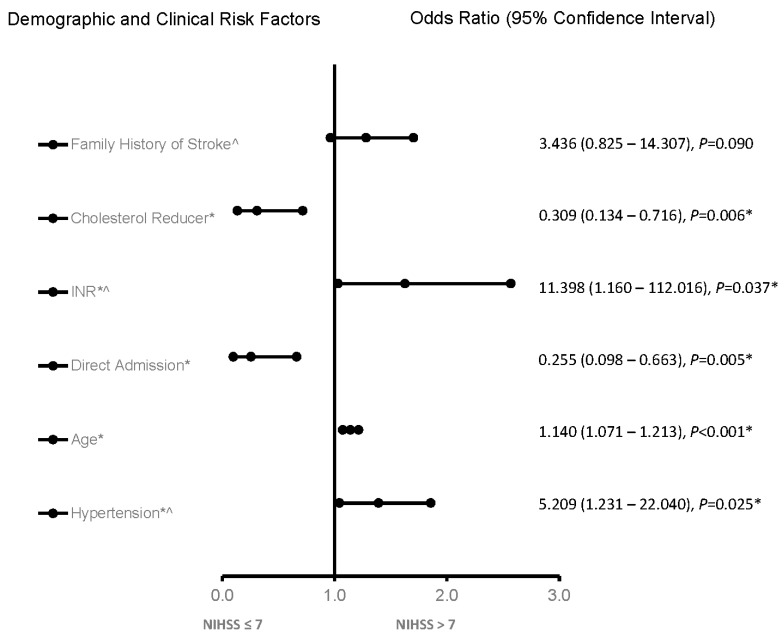
Factors associated with ischemic stroke patients, independent of age, in the telestroke database. Adjusted OR < 1 denotes factors associated with improving neurologic function or NIH ≤ 7. Hosmer–Lemeshow test: *p* = 0.767; Cox and Snell: R2 = 0.268. The overall classified percentage of 75.4% was applied to check the fitness of the logistic regression model. * indicates statistical significance (*p* < 0.05) with a 95% confidence interval, ^ Indicates that data were modified by taking the 5th square root.

**Figure 2:**
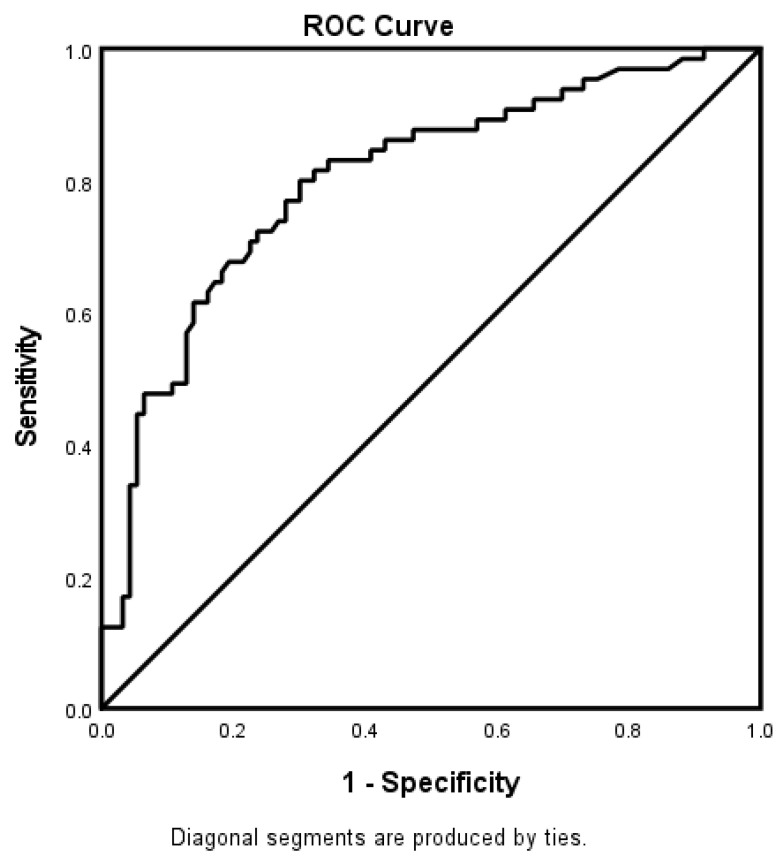
ROC curve associated with predicting stroke severity for acute ischemic stroke patients, independent of age, in the telestroke database. The area under the ROC analysis curve (AUC) values indicate better discrimination of the score for the measured outcome. Therefore, the classification table (overall correctly classified percentage = 75.4%) and the area under the ROC curve (AUC = 0.802, 0.732–0.873) were applied to check the model fitness.

In the adjusted analysis, irrespective of the age category, INR, increasing age, and hypertension were associated with worsening neurological functions, while taking a cholesterol reducer and direct admission were associated with improving neurologic functions in the whole stroke population (Figure 1). The ROC curve for the predictive power of the regression model is presented in Figure 2. The discriminating capability of the model was strong, as shown by the ROC curve, with an area under the curve (AUROC) of 0.802 (95% CI = 0.732–0.873, *p* < 0.001). 

**Figure 3:**
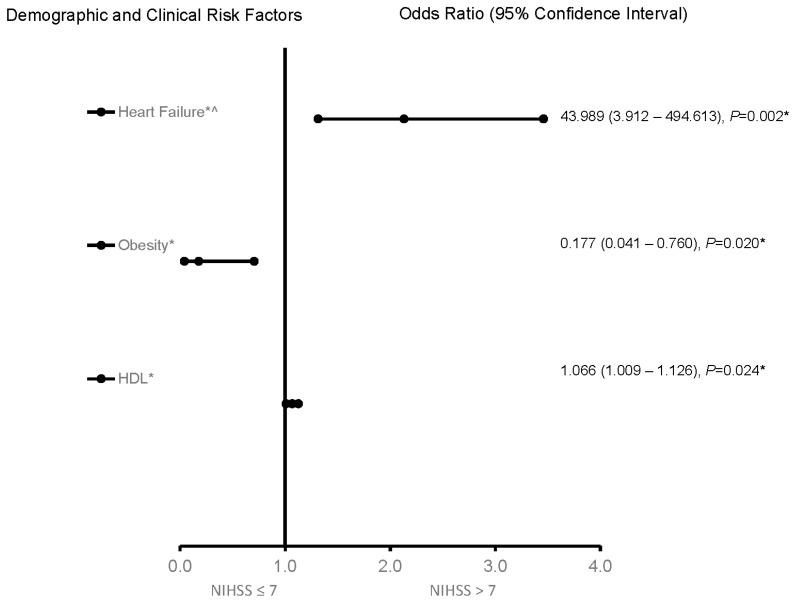
Factors associated with stroke severity for 65–74-year-old patients. OR > 1 denotes factors associated with having an NIHSS score > 7 or worsening neurologic functions. Hosmer–Lemeshow test: *p* = 0.543; Cox and Snell: R2 = 0.235. The overall classified percentage of 84.9% was applied to check the fitness of the logistic regression model. * indicates statistical significance (*p* < 0.05) with a 95% confidence interval, ^ Indicates that data were modified by taking the 5th square root.

**Figure 4:**
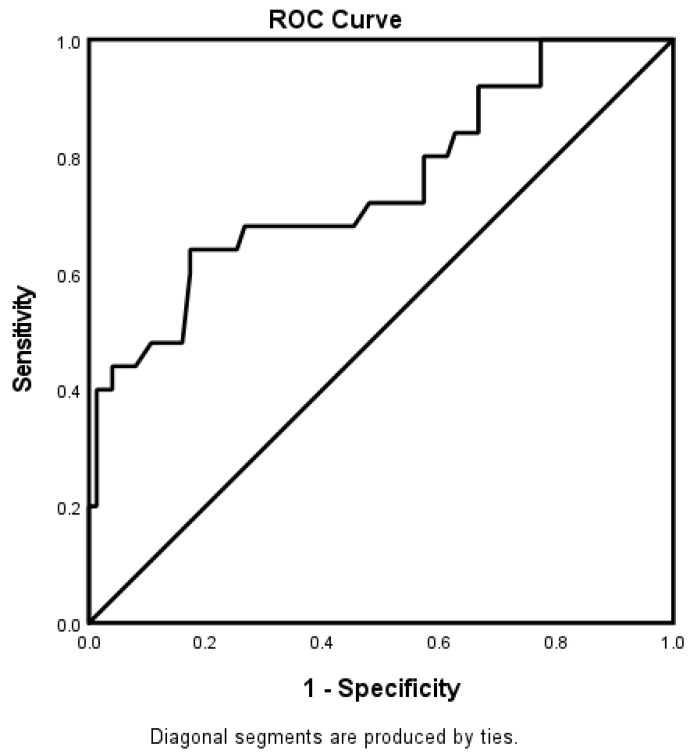
ROC curve associated with stroke severity in 65–74-year-old patients. Hosmer–Lemeshow test: *p* = 0.676; Cox and Snell: R2 = 0.098. The overall classified percentage of 76.3% was applied to check the fitness of the logistic regression model.

**Figure 5:**
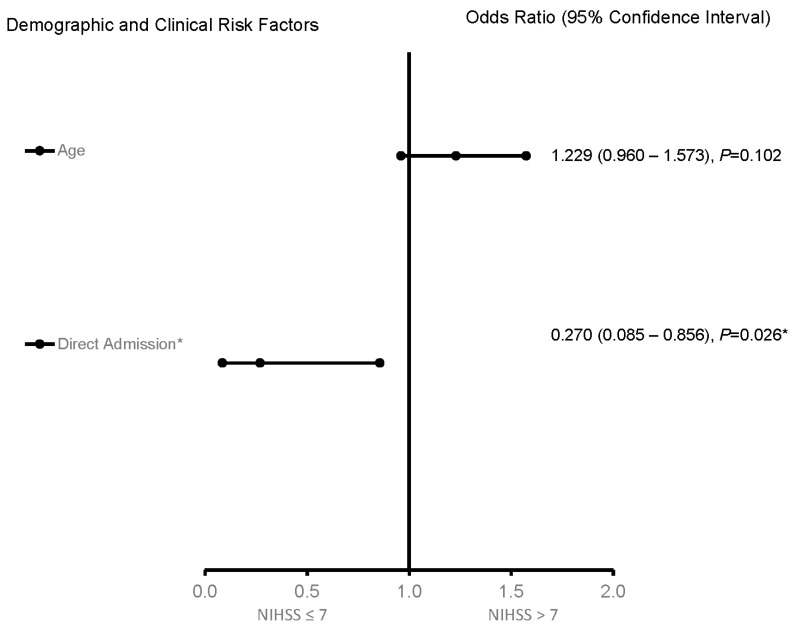
Factors associated with stroke severity for patients ≥ 75 years old in the telestroke database. Adjusted OR > 1 denotes factors associated with having an NIHSS score > 7 or worsening neurologic outcomes. Hosmer–Lemeshow test: *p* = 0.676; Cox and Snell: R2 = 0.098. The overall classified percentage of 76.3% was applied to check the fitness of the logistic regression model. * indicates statistical significance (*p* < 0.05) with a 95% confidence interval.

**Figure 6:**
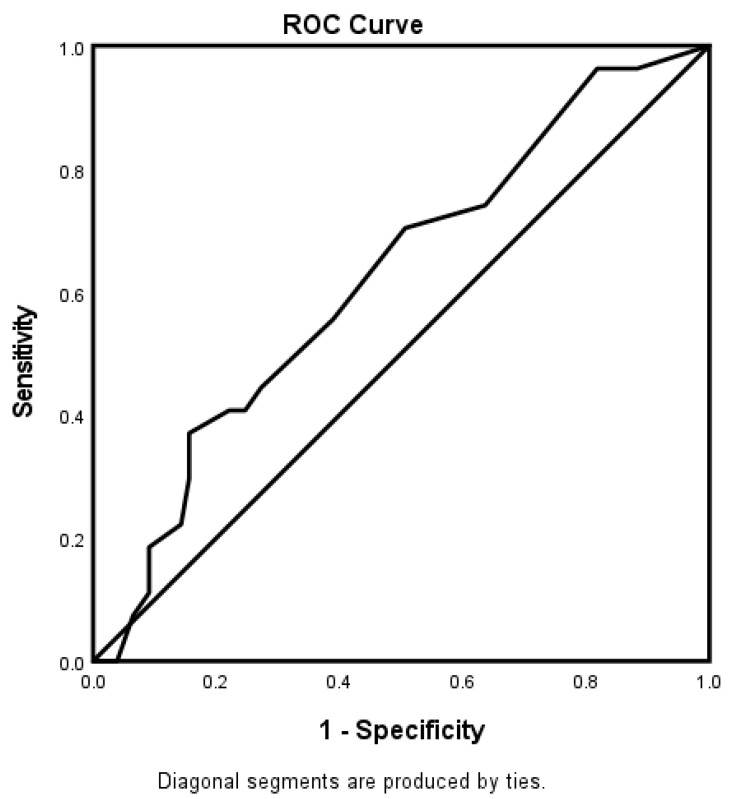
ROC curve associated with predicting stroke severity for patients ≥ 75 years old in the telestroke database. The area under the curve (AUC) values in the ROC analysis indicate discrimination of the score for the measured outcome. The classification table (overall correctly classified percentage = 76.3%) and the area under the ROC curve (AUC = 0.622, 0.502–0.743) were applied to check the model fitness.

For the 65–74-year-old category (Figure 3), heart failure and high HDL were associated with worsening neurologic functions. Obese AIS patients were more likely to be associated with improving neurologic functions. The predictive power of the logistic regression was moderately strong (Figure 4). The AUROC was 0.750 (95% CI = 0.631–0.869, *p* < 0.001). For the ≥75-year-old age category, direct admission for treatment was associated with improving neurological functions, while increasing age was associated with worsening neurological functions (Figure 5). Figure 6 shows the discriminating capability of the model through an ROC curve. The AUROC was strong (0.622, 95% CI = 0.502–0.743, *p* = 0.060). 

## 4. Discussion

Age is a significant risk factor for stroke, and an estimated 75% of strokes occur in people > 65 years old [25]. While aged patients present higher mortality and poorer quality of life after stroke than younger patients, approximately 50% of all strokes occur in people over age 75 [5]. Moreover, the incidence of stroke is double in adults over the age of 75 years [3], and those ≥ 75 years experience more hospitalizations and higher mortality related to stroke [4]. Therefore, given the increasing longevity of human populations with different multimorbidities [26], identifying risk factors of stroke in patients aged 65–74 years old, when about 75% of all strokes occur, and ≥75 years, when the incidence of stroke is double and more hospitalizations and higher mortality occur [15], will be vital to guiding improvements in the quality of care for older AIS patients. 

In a population of AIS patients, we found that more patients aged 65–74 were treated in the telestroke network compared with those aged ≥75. Moreover, patients aged 65–74 years old with worsening neurological functions presented with higher rates of heart failure, while those aged ≥75 years old with declining neurological functions were less likely to be older to taking a cholesterol reducer. Irrespective of the age category, the international normalized ratio (INR), increasing age, and hypertension were associated with worsening neurological functions, while taking a cholesterol reducer was associated with improvements following the univariate analysis. Direct admission was associated with improved neurologic functions in the adjusted analysis for the whole AIS population. While the use of a cholesterol reducer, INR, increasing age, and hypertension were all significant for improving or worsening neurologic functions, the effects of these factors were attenuated in the adjusted analysis for the 65–74-year-old age category. Although the effects of the previous history of congestive heart failure and HDL were associated with worsening neurologic functions, obesity was more likely to be associated with an improvement in neurologic functions in the 65–74-year-old category of AIS patients treated in the telestroke database. 

Cardioembolic stroke is a significant cause of ischemic stroke in elderly patients [27]. Several pathophysiologic mechanisms of CHF that could cause an AIS have been described [28], and the most frequently recognized reason for an AIS in patients with CHF is thrombus formation due to left ventricular (LV) hypokinesia [29]. Therefore^,^ CHF is directly linked with increased thrombus formation and stroke risk. Moreover, CHF represents the most common reason for hospitalization in patients older than 65 [30]. This finding supports our current association of CHF with poor neurologic functions in the 65–74-year-old AIS patients treated in the telestroke network. In general, stroke-related mortality rates are significantly higher in AIS patients with CHF than those without CHF. In the addition, cardioembolic stroke in patients with CHF is also linked to atrial fibrillation (AF). AF is the most common cause of thromboembolic complications. The risk of suffering a thromboembolic complication is contingent on the accompanying cardiac risk factors and the patient’s age. AF is reported to be prevalent in over 3% of the adult population and causes thromboembolic disease, mainly ischemic stroke [28,31]. The risk of stroke and death in patients with AF is strongly associated with age and associated comorbidities [32]. The prevalence of AF is reported to increase by two-fold with each decade of age; therefore, age is an important risk factor for the development of AF [29]. The risk of stroke and thromboembolism is increased in AF, but this risk is not consistent in all patients and is dependent on the presence of several stroke risk factors in the individual patients, including congestive heart failure, hypertension, age ≥ 75, diabetes, and stroke/transient cerebral ischemia [33].

While the complex interplay between CHF and AIS is clinically relevant, developing a management strategy for the care for 65–74-year-old patients with a history of CHF will help improve functioning in this age category of AIS patients.

The concept that “lower cholesterol levels are associated with better functions” has been proposed to help prevent cardiovascular diseases [34]. However, an inconsistent association in the metabolic significance of lipids with stroke has been reported in several studies. For example, some studies found no association [33], while others found good associations [35] for HDL with stroke severity (NIHSS) and stroke outcomes in AIS. Our current study predicted worsening neurologic outcomes in AIS patients with HDL. One possible explanation is that AIS increases the proportion of dysfunctional HDL with abundant myeloperoxidase and α1-antitrypsin [36]. This dysfunctional HDL exerts a stressful effect on the endothelial cells, resulting in a poor association between HDL and stroke outcomes [37]. Although existing studies support an inverse relationship between the HDL concentration (HDL-C) and cardiovascular disease (CVD) [38], the specific relationships between stroke and both HDL-C and its subclasses are also reported to be very complicated. HDL fragments are remarkably heterogeneous in size, density, composition, and surface charge [39]. In addition, HDL subclasses differ in their ability to promote cholesterol efflux, which is the initial stage in the mechanism associated with reverse cholesterol transport [40]. While acute ischemic stroke patients are known to facilitate an increase in small HDL particles [41], some experimental studies reported that higher HDL-C levels were associated with lower stroke risk [42]. Other studies found no relation [43], and some found an increased risk [44]. The latter supports our current finding of an association of HDL with worsening neurologic functions in AIS. Therefore, more studies are necessary to determine the relationships between the different classes of HDL, AIS severity, and stroke outcomes. This was identified as a significant predictor of stroke severity in 65–74-year-old AIS patients treated in the telestroke network.

We observed that obesity, as a risk factor, was more likely to improve neurologic functions in the 65–74-year-old age category of AIS patients. Obesity is a risk factor for ischemic stroke; its effect on treatment outcomes in AIS patients is unclear [45]. The description of obesity categories is based on *body mass index* (BMI), and a U-shaped association between BMI and stroke has been reported [46]. However, other studies [47,48] support the paradoxical concept of relative longevity in obese patients with stroke. It is also possible that the obesity paradox may exist in the 65–74-year-old category of AIS patients and improve neurologic functions in this age category of AIS patients with excess body weight treated in the telestroke network. Our finding of an association between obesity and improving neurologic functions is similar to other studies in stroke [49], coronary artery disease, and congestive heart disease [50]. This possibility needs to be interpreted cautiously, especially since we used an analytical approach that did not provide the opportunity for the randomization of our data collection. Therefore, a causal relationship cannot be established. Therefore, well-designed randomized controlled trials analyzing the effects of weight reduction on stroke risk in the 65–74-year-old category of AIS patients will be necessary in future studies.

We found that AIS patients ≥ 75 years old that were directly admitted for treatment in the telestroke setting were associated with improving neurologic functions. Other studies [31,51] support this result, indicating that direct admission leads to a shorter onset to needle time and improves patient functions in AIS patients treated in the telestroke network. Therefore, one method for decreasing the time and increasing the efficient evaluation and administration of rtPA to AIS patients is system-based telestroke care [32]. Therefore, our data contribute to the existing literature stating that the telestroke evaluation of ≥75-year-old AIS patients leads to improved neurologic functions via the facilitation of direct admissions.

We observed that AIS patients ≥ 75 years old with increasing age were more likely to have worsening neurologic functions. Aging is the most vital nonmodifiable risk factor for AIS, and aged stroke patients present higher mortality and poorer functional recovery than their young AIS patients [31]. Notably, patients’ ages are known to modify the effects of other comorbidities on AIS, and nearly two thirds of AISs are proposed to occur after age 75 [51]. While the future of stroke is proposed to include a substantial increase in stroke events, an increasing percentage of stroke events is also projected to involve patients over 75 years old. Unlike in younger adults, where the evidence for primary and secondary stroke prevention is well established and supported by robust randomized clinical trial data, the evidence base is less clear in older adults, especially those aged ≥75 years. Our current finding that increasing age was more likely to be associated with worsening neurologic functions in AIS patients ≥ 75 years old indicates that it may be necessary to develop effective stroke prevention and treatment approaches to ameliorate comorbidity among ≥75-year-old stroke patients to achieve optimal clinical outcomes for the care of stroke patients treated in the telestroke network.

### Limitations and Strengths

Our study had limitations. First, our study was limited to elderly 65–74- and ≥75-year-old patients in the telestroke network, and there is a concern regarding the applicability of these findings to younger patient populations. Second, some specific HDL-C subclasses were not measured, and we could not verify the operating mechanisms to explain the identified risk associations of HDL and AIS. Therefore, more studies are necessary to determine the relationships between different classes of HDL, AIS severity, and stroke outcome. This has been identified as a significant predictor of stroke severity in elderly populations. Moreover, we conducted a retrospective data analysis and not a randomized controlled trial. Therefore, the possibility that an obesity paradox may exist in the 65–74-year-old category needs to be interpreted cautiously, especially since a causal relationship cannot be established. Patients in this study were not divided into groups according to the pathogenetic type of stroke (atherothrombotic, cardioembolic, and lacunar), especially since the pathogenesis and risk factors differ depending on the pathogenetic type of stroke. On the other hand, the strength of our study was using data from a regional stroke center that provided quality treatment in the telestroke network. Therefore, this study was well equipped to determine the stroke severity among AIS patients stratified into 65–74- and ≥75-year-old categories in the telestroke network. An essential contribution of this study to the existing literature in telestroke neurology research is our finding that NIHSS can be used to stratify risk factors that contribute to stroke severity in the aging population treated in the telestroke network.

## 5. Conclusions

Aging is a crucial risk factor for AIS, and aged stroke patients have higher morbidity and worse functional recovery than young patients. About 75% of all strokes are predicted to occur in people > 65 years old [25], and adults > 75 years of age experience more hospitalizations, higher mortality associated with stroke, and about 50% of all strokes [4]. This study observed differences in stroke risk factor profiles for the 65–74- and ≥75-year-old age categories. Most importantly, the findings from this study reveal specific risk factors that can be managed to improve care in older stroke patients treated in the telestroke network.

## Figures and Tables

**Table 1:** Comparison of demographics and clinical characteristics of acute ischemic stroke patients in the telestroke database. Demographic and clinical characteristics of ischemic stroke patients were divided by age (65–74 years or ≥75 years old). Results for continuous variables are presented as means ± SDs, while discrete data are presented as percentage frequencies. Pearson’s chi-square test was used to compare differences between demographic and clinical characteristics in 65–74-year-old and ≥75-year-old patients.

Characteristic	Age 65–74 Years Old	Age ≥ 75 Years Old	
Number of Patients	104	119	*p*-Value
Age: Mean ± SD	69.81 ± 2.43	81.61 ± 5.48	<0.001 *^,b^
Race: No. (%)			
White	90 (86.5)	100 (84.0)	0.865
Black	12 (11.5)	16 (13.4)	
Other	2 (1.9)	3 (2.5)	
Gender: No. (%)			
Female	52 (50.0)	70 (58.8)	0.187
Male	52 (50.0)	49 (41.2)	
Hispanic Ethnicity: No. (%)	2 (1.9)	1 (0.8)	0.484
BMI: Mean ± SD	28.99 ± 5.74	27.22 ± 4.78	0.014 *^,b^
Medical History: No. (%)			
Atrial Fibrillation	9 (8.7)	34 (28.6)	<0.001 *^,a^
Coronary Artery Disease	36 (34.6)	49 (41.2)	0.314
Carotid Artery Stenosis	4 (3.8)	11 (9.2)	0.108
Depression	6 (5.8)	17 (14.3)	0.037 *^,a^
Diabetes	46 (44.2)	47 (39.5)	0.474
Drugs or Alcohol	1 (1.0)	1 (0.8)	0.924
Dyslipidemia	66 (63.5)	68 (57.1)	0.336
Stroke Family History	9 (8.7)	9 (7.6)	0.765
Heart Failure	8 (7.7)	20 (16.8)	0.040 *^,a^
Hormonal Replacement Therapy	0 (0.0)	2 (1.7)	0.184
Hypertension	84 (80.8)	106 (89.1)	0.081
Migraine	2 (1.9)	0 (0.0)	0.129
Obesity	45 (43.3)	50 (42.0)	0.850
Previous Stroke	25 (24.0)	25 (21.0)	0.588
Previous TIA (>24 h)	7 (6.7)	19 (16.0)	0.032 *^,a^
Prosthetic Heart Valve	1 (1.0)	1 (0.8)	0.924
Peripheral Vascular Disease	6 (5.8)	14 (11.8)	0.118
Chronic Renal Disease	5 (4.8)	7 (5.9)	0.723
Sleep Apnea	1 (1.0)	4 (3.4)	0.227
Smoker	21 (20.2)	8 (6.7)	0.003 *^,a^
Medication History: No. (%)			
HTN Medication	79 (76.0)	98 (82.4)	0.239
Cholesterol Reducer	57 (54.8)	57 (47.9)	0.303
Diabetes Medication	32 (30.8)	37 (31.1)	0.958
Antidepressant	8 (7.7)	20 (16.8)	0.040 *^,a^
Initial NIHSS Score: No. (%)			
0–9	79 (79.0)	64 (61.0)	0.039 *^,a^
10–14	9 (9.0)	14 (13.3)	
15–20	7 (7.0)	17 (16.2)	
21–25	5 (5.0)	10 (9.5)	
Mean ± SD	6.58 ± 7.64	10.63 ± 8.90	<0.001 *^,b^
Lab values: Mean ± SD			
Total cholesterol	165.03 ± 47.18	168.35 ± 44.75	0.601
Triglycerides	136.94 ± 66.11	123.05 ± 94.75	0.224
HDL	40.09 ± 10.48	45.01 ± 14.09	0.004 *^,b^
LDL	100.06 ± 38.22	101.39 ± 935.38	0.794
Lipids	6.53 ± 1.71	6.36 ± 1.53	0.464
Blood Glucose	140.01 ± 61.39	134.85 ± 66.63	0.556
Serum Creatinine	1.08 ± 0.56	1.15 ± 0.53	0.296
INR	1.05 ± 0.17	1.10 ± 0.28	0.147
Vital Signs: Mean ± SD			
Heart Rate	75.90 ± 15.53	80.19 ± 15.69	0.042 *^,b^
Systolic Blood Pressure	152.37 ± 24.10	152.65 ± 24.11	0.931
Diastolic Blood Pressure	78.93 ± 16.30	78.99 ± 19.77	0.981
Ambulation Status Prior to Event: No. (%)			
Ambulated Independently	101 (97.1)	101 (84.9)	0.020 *^,a^
Ambulated with Assistance	1 (1.0)	4 (3.4)	
Unable to Ambulate	1 (1.0)	7 (5.9)	
Not Documented	1 (1.0)	7 (5.9)	
Ambulation Status on Admission: No. (%)			
Ambulated Independently	25 (24.0)	15 (12.6)	0.023 *^,a^
Ambulated with Assistance	30 (28.8)	35 (29.4)	
Unable to Ambulate	24 (23.1)	47 (39.5)	
Not Documented	25 (24.0)	22 (18.5)	
Ambulation Status on Discharge: No. (%)			
Ambulated Independently	59 (56.7)	32 (26.9)	<0.001*^,a^
Ambulated with Assistance	27 (26.0)	43 (36.1)	
Unable to Ambulate	8 (7.7)	33 (27.7)	
Not Documented	10 (9.6)	11 (9.2)	
rtPA received: No. (%)	70 (67.3)	80 (67.2)	0.990
Emergency Department	28 (26.9)	25 (21.0)	0.301
Direct Admission	76 (73.1)	94 (79.0)	
Improved Ambulation: No. (%)	51 (54.3)	41 (37.6)	0.018 *^,a^
NIHSS > 7: No. (%)	27 (26.0)	61 (54.5)	<0.001 *^,a^
Diastolic Blood Pressure ≥ 80 mmHg	46 (44.2)	54 (45.4)	0.864

Notes: ^a^ Pearson’s chi-squared test; ^b^ Student’s *t*-test; * *p*-value < 0.05.

**Table 2:** Comparison of demographics and clinical characteristics of acute ischemic stroke patients in the telestroke database based on age using NIHSS scores. The demographic and clinical characteristics of ischemic stroke patients in the telestroke database were stratified by age (65–74 years old or ≥75 years old). Results for continuous variables are presented as means ± SDs, while discrete data are presented as percentage frequencies. Pearson’s chi-square test was used to compare differences between demographic and clinical characteristics in groups with NIHSS scores greater than 7 in the telestroke database based on age (65–74 years old or ≥75 years old).

	Age 65–74 Years Old			Age ≥75 Years Old		
Characteristic	NIHSS ≤ 7	NIHSS > 7		NIHSS ≤ 7	NIHSS > 7	
Number of Patients	77	27	*p*-Value	51	61	*p*-Value
Age: Mean ± SD	69.71 ± 2.54	70.07 ± 2.13	0.511	80.59 ± 4.45	82.62 ± 6.01	0.042 *^,b^
Race: No. (%)						
White	67 (87.0)	23 (85.2)	0.592	46 (90.2)	48 (78.7)	0.146
Black	8 (10.4)	4 (14.8)		5 (9.8)	10 (16.4)	
Other	2 (2.6)	0 (0.0)		0 (0.0)	3 (4.9)	
Gender: No. (%)						
Female	39 (50.6)	13 (48.1)	0.823	28 (54.9)	38 (62.3)	0.428
Male	38 (49.4)	14 (51.9)		23 (45.1)	23 (37.7)	
Hispanic Ethnicity: No. (%)	2 (2.6)	0 (0.0)	0.398	0 (0.0)	1 (1.6)	0.358
BMI: Mean ± SD	29.20 ± 5.31	28.37 ± 6.95	0.531	27.83 ± 4.86	26.56 ± 4.52	0.160
Medical History: No. (%)						
Atrial Fibrillation	5 (6.5)	4 (14.8)	0.186	10 (19.6)	22 (36.1)	0.055
Coronary Artery Disease	27 (35.1)	9 (33.3)	0.871	21 (41.2)	26 (42.6)	0.877
Carotid Artery Stenosis	4 (5.2)	0 (0.0)	0.227	4 (7.8)	7 (11.5)	0.520
Depression	4 (5.2)	2 (7.4)	0.671	8 (15.7)	8 (13.1)	0.699
Diabetes	37 (48.1)	9 (33.3)	0.185	22 (43.1)	22 (36.1)	0.445
Drugs or Alcohol	1 (1.3)	0 (0.0)	0.552	0 (0.0)	1 (1.6)	0.358
Dyslipidemia	51 (66.2)	15 (55.6)	0.321	28 (54.9)	35 (57.4)	0.793
Stroke Family History	8 (10.4)	1 (3.7)	0.288	3 (5.9)	6 (9.8)	0.443
Heart Failure	2 (2.6)	6 (22.2)	<0.001 *^,a^	6 (11.8)	11 (18.0)	0.357
Hormonal Replacement Therapy	0 (0.0)	0 (0.0)		1 (2.0)	1 (1.6)	0.898
Hypertension	62 (80.5)	22 (81.5)	0.913	42 (82.4)	57 (93.4)	0.068
Migraine	2 (2.6)	0 (0.0)	0.398	0 (0.0)	0 (0.0)	
Obesity	37 (48.1)	8 (29.6)	0.096	23 (45.1)	25 (41.0)	0.661
Previous Stroke	20 (26.0)	5 (18.5)	0.435	9 (17.6)	13 (21.3)	0.627
Previous TIA (>24 h)	7 (9.1)	0 (0.0)	0.105	8 (15.7)	10 (16.4)	0.919
Prosthetic Heart Valve	1 (1.3)	0 (0.0)	0.552	0 (0.0)	1 (1.6)	0.358
Peripheral Vascular Disease	3 (3.9)	3 (11.1)	0.166	6 (11.8)	8 (13.1)	0.830
Chronic Renal Disease	5 (6.5)	0 (0.0)	0.175	2 (3.9)	5 (8.2)	0.352
Sleep Apnea	1 (1.3)	0 (0.0)	0.522	3 (5.9)	1 (1.6)	0.228
Smoker	16 (20.8)	5 (18.5)	0.801	2 (3.9)	6 (9.8)	0.226
Medication History: No. (%)						
HTN Medication	59 (76.6)	20 (74.1)	0.790	38 (74.5)	53 (86.9)	0.095
Cholesterol Reducer	45 (58.4)	12 (44.4)	0.209	31 (60.8)	22 (36.1)	0.009 *^,a^
Diabetes Medication	24 (31.2)	8 (29.6)	0.881	18 (35.2)	17 (27.9)	0.399
Antidepressant	6 (7.8)	2 (7.4)	0.949	7 (13.7)	12 (19.7)	0.404
Lab values: Mean ± SD						
Total cholesterol	162.23 ± 46.27	173.44 ± 49.81	0.306	165.12 ± 45.35	169.12 ± 45.35	0.643
Triglycerides	138.25 ± 64.72	133.00 ± 71.34	0.733	129.18 ± 85.99	116.50 ± 103.13	0.493
HDL	38.71 ± 10.44	44.24 ± 9.66	0.022 *^,b^	45.16 ± 15.68	44.76 ± 12.96	0.884
LDL	98.71 ± 38.11	104.12 ± 39.05	0.542	97.86 ± 34.70	102.76 ± 35.53	0.472
Lipids	6.58 ± 1.76	6.34 ± 1.54	0.551	6.35 ± 1.39	6.39 ± 1.68	0.892
Blood Glucose	141.01 ± 66.98	137.12 ± 42.27	0.782	135.92 ± 63.37	137.58 ± 71.71	0.899
Serum Creatinine	1.09 ± 0.61	1.04 ± 0.40	0.676	1.19 ± 0.56	1.13 ± 0.50	0.540
INR	1.04 ± 0.12	1.09 ± 0.28	0.289	1.03 ± 0.12	1.13 ± 0.33	0.089
Vital Signs: Mean ± SD						
Heart Rate	76.34 ± 14.17	74.67 ± 19.13	0.633	78.14 ± 15.33	82.31 ± 16.03	0.164
Systolic Blood Pressure	151.03 ± 22.40	156.19 ± 28.53	0.341	154.51 ± 23.58	153.49 ± 24.22	0.823
Diastolic Blood Pressure	78.18 ± 16.81	81.07 ± 14.82	0.430	79.76 ± 20.75	79.77 ± 19.09	0.999
Ambulation Status Prior to Event: No. (%)						
Ambulated Independently	76 (98.7)	25 (92.6)	0.105	47 (92.2)	50 (82.0)	0.179
Ambulated with Assistance	1 (1.3)	0 (0.0)		0 (0.0)	3 (4.9)	
Unable to Ambulate	0 (0.0)	1 (3.7)		3 (5.9)	3 (4.9)	
Not Documented	0 (0.0)	1 (3.7)		1 (2.0)	5 (8.2)	
Ambulation Status on Admission: No. (%)						
Ambulated Independently	25 (32.5)	0 (0.0)	<0.001 *^,a^	13 (25.5)	1 (1.6)	<0.001 *^,a^
Ambulated with Assistance	26 (33.8)	4 (14.8)		23 (45.1)	10 (16.4)	
Unable to Ambulate	2 (2.6)	22 (81.5)		5 (9.8)	38 (62.3)	
Not Documented	24 (31.2)	1 (3.7)		10 (19.6)	12 (19.7)	
Ambulation Status on Discharge: No. (%)						
Ambulated Independently	54 (70.1)	5 (18.5)	<0.001 *^,a^	24 (47.1)	7 (11.5)	<0.001 *^,a^
Ambulated with Assistance	18 (23.4)	9 (33.3)		22 (43.1)	19 (31.1)	
Unable to Ambulate	2 (2.6)	6 (22.2)		5 (9.8)	25 (41.0)	
Not Documented	3 (3.9)	7 (25.9)		0 (0.0)	10 (16.4)	
rtPA Administration	52 (67.5)	18 (66.7)	0.934	33 (64.7)	46 (75.4)	0.216
Emergency Department	17 (22.1)	11 (40.7)	0.060	8 (15.7)	15 (24.6)	0.245
Direct Admission	60 (77.9)	16 (59.3)		43 (84.3)	46 (75.4)	
Improved Ambulation: No. (%)	38 (51.4)	13 (65.0)	0.277	19 (37.3)	22 (42.3)	0.600
Diastolic Blood Pressure ≥ 80 mmHg	33 (42.9)	13 (48.1)	0.634	22 (43.1)	30 (49.2)	0.523

Notes: ^a^ Pearson’s chi-squared test; ^b^ Student’s *t*-test; * *p*-value < 0.05.

## Data Availability

The retrospective datasets are available upon request from the corresponding author of this manuscript.

## Abbreviations

Adjusted OR: adjusted odds ratio; atrial fibrillation: Afib, CI; confidence interval; BMI: body mass index; CHF: congestive heart failure; CI: confidence interval; IRB: institutional review board. INR: international normalized ratio; LDL-C: low-density lipoprotein cholesterol; HDL-C: high-density lipoprotein cholesterol; rtPA: recombinant tissue plasminogen; TC: total cholesterol; TG: triglyceride, AIS: acute ischemic stroke; NIHSS: National Institute of Health Stroke Scale; MRI: magnetic resonance imaging; CT: computer tomography; MCA: middle cerebral artery; CAD: coronary artery disease; HRT: hormone replacement therapy; TIA: transient ischemic attack; PVD: peripheral vascular disease; ROC: receiver operating curve; INR: international normalized ratio; HRV: heart rate variability; TP: total power; LF: low frequency; HF: high-frequency domains.
